# Impact and safety of perineal massage in late pregnancy on delivery outcomes among primiparous women: a propensity score matching analysis

**DOI:** 10.1186/s12884-025-08452-9

**Published:** 2025-11-26

**Authors:** Jiaojiao Zhang, Fengcheng Cai, Yuanyuan Li, Mengyan Xu

**Affiliations:** 1 Hangzhou Women’s Hospital, Hangzhou, 310008 China; 2 Hangzhou Ninth People’s Hospital, Hangzhou, 311225 China; 3 Zhejiang Chinese Medical University, Hangzhou, 310053 China

**Keywords:** Primigravida, Late pregnancy, Perineal massage, Propensity score matching, Labor outcomes, Safety, Influencing factors

## Abstract

**Background:**

Primiparas face a higher risk of perineal injury, which may adversely affect maternal and infant health. Perineal massage has been proposed as an intervention to improve birth outcomes, but evidence of its effectiveness remains inconsistent, and concerns about its safety persist. This study aimed to evaluate the effects and safety of late-pregnancy perineal massage on birth outcomes in primiparas using propensity score matching.

**Methods:**

A retrospective cohort study was conducted involving 914 primigravid women who received routine perineal massage at a tertiary A-level maternity hospital in Hangzhou from January to December 2024, comprising the massage group. Using propensity score matching (PSM), 914 primigravid women without perineal massage during the same period were matched 1:1 from a pool of 3,104 eligible participants, forming the control group, for a total of 1,828 cases. Differences between the two groups were compared in terms of perineal integrity, episiotomy rate, degree of perineal laceration, duration of labor, postpartum hemorrhage, premature rupture of membranes, preterm birth, and neonatal asphyxia. Related influencing factors were further analyzed.

**Results:**

The massage group showed a significantly higher rate of perineal integrity compared with the control group (2.2% vs. 0.5%, *P* = 0.02) and a lower episiotomy rate (16.3% vs. 21.2%, *P* = 0.02). The incidence of second-degree laceration was lower (22.9% vs. 24.7%, *P* = 0.02), and the second stage of labor was shorter (1.27 h vs. 1.42 h, *P* = 0.001). The rates of premature rupture of membranes (22.4% vs. 27.2%, *χ²*=7.567, *P* = 0.006) and postpartum hemorrhage (293 ± 130 ml vs. 306 ± 138 ml, *P* = 0.038) were also slightly lower in the massage group. No significant differences were observed between groups in preterm birth or neonatal asphyxia (*P* > 0.05). The number of perineal massages was negatively correlated with both the degree of perineal injury (*OR* = 0.723, 95% *CI*: 0.670–0.780, *P* < 0.001) and the duration of the second stage of labor (Spearman’s ρ=-0.303, *P* < 0.001). Optimal perineal protection was observed with approximately seven massage sessions (IQR: 3–8, *P* < 0.001).

**Conclusion:**

The study find that regular perineal massage is effective in reducing the risk of perineal injury, shortening the duration of the second stage of labor without causing preterm labor, preterm rupture of membranes, or alteration of neonatal health, and has a favorable safety profile. The frequency of clinical massage (2–3 times per week, cumulative ≥ 7 times) is recommended to protect the perineum, shorten the duration of labor, and optimize labor outcomes.

## Introduction

Delivery outcome primarily refers to the mode of delivery, duration of labor, postpartum hemorrhage, perineal condition, and neonatal health [1]. These outcomes are closely related to both maternal and fetal health and have a direct impact on family and societal well-being. Among primigravid women, labor is typically prolonged, and the risk of perineal injury can reach up to 85% [2]. In addition, they face the potential need for conversion to cesarean Sect [3], which may cause complications such as pain, postpartum hemorrhage, infection, and neonatal asphyxia. These factors significantly affect delivery outcomes and influence decisions regarding the mode of delivery [2]. Therefore, identifying effective interventions to improve labor outcomes in primiparous women has become a key focus in perinatal medicine. Perineal massage, a non-pharmacological intervention performed in late pregnancy, aims to promote local blood circulation in the perineum and enhance the nutrient supply to the perineal tissues through specific professional techniques. This process improves perineal elasticity and resilience, thereby reducing the risk of perineal trauma during childbirth [4]. Theoretically, regular perineal massage facilitates better perineal stretching during labor, allowing smoother fetal passage through the birth canal and improving overall delivery outcomes [5]. However, the precise effects of perineal massage on labor outcomes [4, 6] remain unclear. Furthermore, evidence is inconclusive regarding its safety, particularly concerning potential risks such as premature rupture of membranes, preterm labor, or infection, due to the limited number of large-scale, high-quality case-control studies. Previous research has also been confounded by multiple variables, including maternal age, gestational stage, and fetal size, which have introduced uncertainty and reduced the reliability of findings. Propensity Score Matching (PSM) offers a statistical approach that simulates randomization by balancing confounding variables, making it suitable for evaluating intervention effects in large retrospective studies [7]. Based on this rationale, the present study employed PSM to control for nine confounding factors, including maternal age, height, weight, and gestational weight gain, to systematically evaluate the effectiveness and safety of perineal massage in improving labor outcomes and to provide robust evidence for clinical decision-making.

## Research methods

### Study population

A retrospective cohort study was conducted, including 914 primigravid women who received regular perineal massage at a tertiary A-level obstetrics and gynecology hospital in Hangzhou from January to December 2024, forming the massage group. Using PSM, 914 women were selected from 3,104 primigravid women who did not receive perineal massage and delivered at the same hospital during the same period. Matching was performed based on nine baseline characteristics, including maternal height, pre-pregnancy weight, age, gestational weight gain, weeks of gestation at delivery, neonatal weight, educational level, analgesic method (epidural or non-epidural), and mode of delivery (spontaneous or assisted vaginal), to ensure the comparability between the two groups.

#### Inclusion criteria

① singleton cephalic pregnancies; ② receipt of standardized perineal massage from 36 weeks of gestation (massage group) or no massage (control group); and ③ delivery at the study hospital with complete delivery records.

#### Exclusion criteria

① multiple pregnancies or abnormal fetal presentations (e.g., breech, transverse); ② presence of severe pregnancy complications (e.g. preeclampsia, placental abruption, or poorly controlled gestational diabetes mellitus); ③ contraindications to vaginal delivery such as placenta previa or malpresentation; ④ pregnancy complicated by reproductive tract infections; and ⑤ Conversion to cesarean section.

This study was approved by the Ethics Committee of Hangzhou Women’s Hospital, China (Approval No. [2025] Medical Ethics Audit A-10). All data were collected in accordance with the hospital’s General Terms of Informed Consent, under which patients provided written consent upon admission for the use of their medical data in scientific research. Data were obtained by the hospital’s Information Department through the Electronic Medical Record System (EMRS) in a compliant manner and in accordance with the ethical principles of the Declaration of Helsinki.

### Data collection

With the assistance of the hospital’s Information Department, clinical data of the study participants were obtained from the EMRS and the midwife clinic system. The collected data included general baseline information such as maternal age, pre-pregnancy weight, height, body mass index (BMI), educational level, weeks of gestation (determined by the last menstrual period and early ultrasound), and gestational weight gain. Delivery-related data included mode of delivery (spontaneous or assisted vaginal), perineal condition (intact perineum, episiotomy, or laceration graded I–IV according to the 2015 Royal College of Obstetricians and Gynaecologists [RCOG] criteria); duration of each labor stage (first, second, and third), total labor duration, and postpartum hemorrhage, which was measured using the gravimetric or volumetric method. Additional variables included the occurrence of premature rupture of membranes or preterm labor (delivery before 37 gestational weeks) and neonatal outcomes such as birth weight and Apgar scores at one and five minutes. All data were verified and cross-checked to ensure completeness and accuracy, and data collection complied with the relevant regulations of the Measures for the Administration of Health Data.

### Grouping method

In the massage group, beginning at 36 weeks of gestation, professional midwives with 10 years of clinical experience and certified competence in perineal anatomy, massage techniques, and practical training provided standardized perineal massage in addition to routine prenatal care. Massages were administered two to three times per week, each lasting 8–10 min, until delivery. During the procedure, the mother adopted a comfortable position, such as the lithotomy or semi-recumbent position. After hand hygiene, the midwife applied a water-soluble lubricant and gently stretched the perineum laterally and downward using the index and middle fingers, followed by U-shaped massage movements with pressure tolerable to the mother [6, 8]. Self-massage was excluded to avoid variability in technique, as professional massage [9] has been shown to be more effective. Maternal responses were closely monitored throughout and after the procedure. If symptoms such as increased pain, vaginal discharge, or bleeding occurred, the massage was stopped immediately, appropriate management was provided, and any adverse events were recorded.

**Fig. 1:**
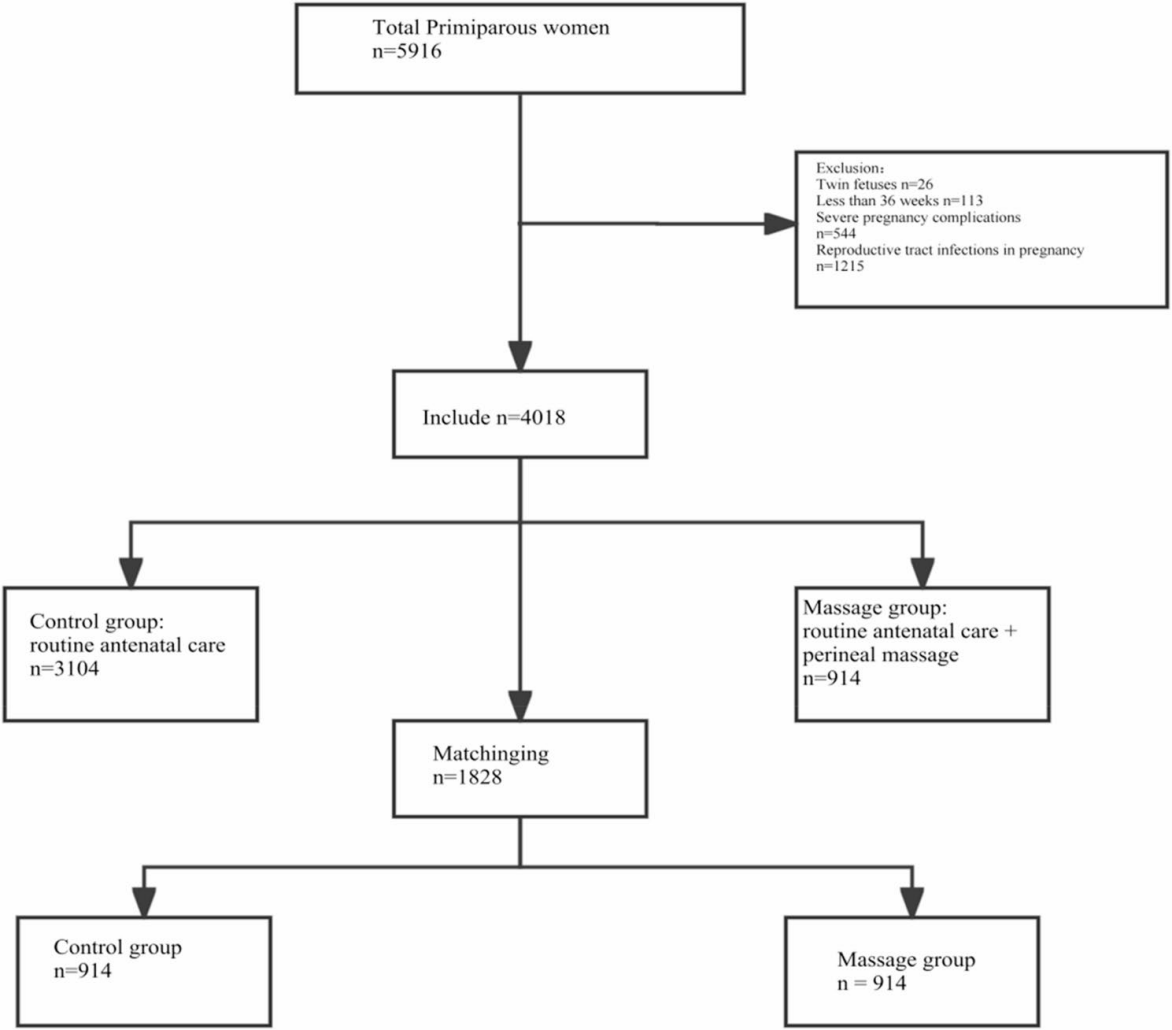
Delivery flow chart

The control group consisted of 914 primigravid women matched by propensity score from a total of 3,104 primigravid women who received routine prenatal care without perineal massage during the same period. The hospital conducts more than 10,000 deliveries annually, and 5,916 primigravid women were included in this study. The detailed delivery process of the hospital is illustrated in Fig. 1.

### Propensity score matching (PSM)

#### Covariate selection

Receipt of perineal massage in late pregnancy was defined as the dependent variable, while maternal age, height, pre-pregnancy weight, educational level, weeks of gestation, gestational weight gain, analgesic method (epidural or non-epidural delivery), mode of delivery, and neonatal weight were included as covariates. These covariates were selected based on their potential influence on labor outcomes and their association with the likelihood of receiving perineal massage.

#### Model construction

A Logistic regression model was applied to calculate the propensity score for each participant. The model was defined as logit(P)=β0 + β1 × 1 + β2 × 2+⋯+βnXn, where P represents the probability of receiving perineal massage, β0 is the intercept, β1-βn are the regression coefficients, and X1-Xn are the corresponding covariates. This model integrated multiple covariates into a composite propensity score that reflected the likelihood of each woman receiving perineal massage.

#### Matching process

**Fig. 2:**
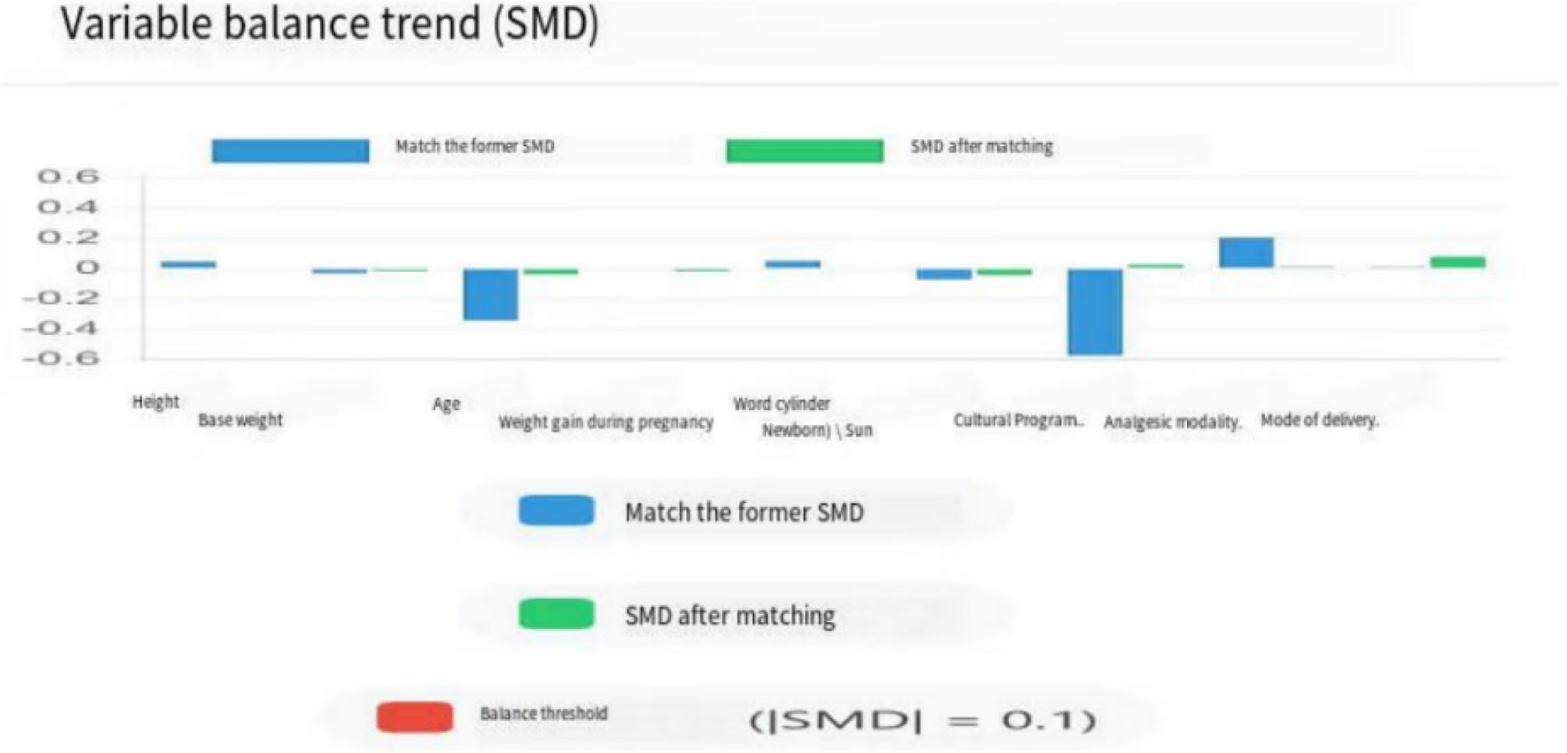
Balance diagram before and after matching

Based on the calculated propensity scores, 1:1 matching was conducted using the caliper matching method, with a caliper width set at 0.2 [7]. For each participant in the massage group, a corresponding non-massage participant with a propensity score difference within 0.2 was selected from the larger pool of primigravid women who did not receive perineal massage, forming the control group. After matching, balance tests were performed on all covariates, and matching effectiveness was evaluated using standardized mean differences (SMD) and related indicators. Before matching, notable imbalances were observed in age (− 0.34), educational level (0.57), and analgesic method (0.20), with the largest imbalance in educational level (SMD = 0.57); most variables exceeded the balance threshold of 0.1. After matching, all SMD absolute values decreased to below 0.08. The SMD for educational level was reduced from 0.57 to 0.02, and for age from − 0.34 to − 0.04, indicating that all variables achieved satisfactory balance below the 0.1 threshold, meeting the statistical requirements for subsequent treatment effect assessment. The balance plot is shown in Fig. 2.

### Observation indicators

#### Delivery outcome indicators

The rates of perineal integrity, episiotomy, and the distribution of perineal laceration degrees, as well as the duration of labor and neonatal birth weight, were compared between the two groups.

#### Safety indicators

Azón E et al. [8] in “Update on the Effectiveness and Evidence-Based Evidence of Antenatal Perineal Massage” reported that perineal massage is safe, with no serious adverse events, tissue damage, clinically significant perineal or vaginal tears, hematomas, or persistent bleeding following the procedure. No cases of fever, abnormal discharge, or pelvic inflammatory reactions occurred within 72 h after massage, and it did not induce preterm labor or increase the frequency of regular uterine contractions. Therefore, this study primarily compared the two groups in terms of severe perineal lacerations (Grade III/IV), postpartum hemorrhage, premature rupture of membranes, preterm labor, neonatal Apgar scores at one and five minutes, and the occurrence of increased pain, vaginal discharge, or bleeding during and after massage.

### Statistical methods

For data description, continuous variables with a normal distribution were presented as mean ± standard deviation (_x±s), while those with a skewed distribution were expressed as median and interquartile range [M (IQR)]. Categorical variables were described as frequency and percentage [*n* (%)]. For between-group comparisons, the independent samples t -test was used for normally distributed continuous variables, and the Kruskal–Wallis H test was applied for non-normally distributed data. The chi-square test (*χ*^*2*^) was used for categorical variables, and Fisher’s exact test was employed when the expected frequency was less than 5. Perineal laceration severity, as an ordered categorical variable (classified as Grades I–IV according to the RCOG criteria), was analyzed using multivariable ordered logistic regression to assess the relationship between the number of massages and laceration severity. Results were reported as odds ratios (*OR*) with 95% confidence intervals (95% *CI*), adjusted for potential confounders including maternal age, pre-pregnancy BMI, neonatal weight, and mode of delivery. The association between the number of massages and the duration of the second stage of labor was analyzed using multiple linear regression, with age, neonatal weight, and mode of delivery included as covariates. All statistical analyses were performed using SPSS version 27.0, with a two-sided *α* level of 0.05. A *P* value of less than 0.05 was considered statistically significant.

## Results

### Results before and after matching of basal data

A total of 5,916 primigravid women were included in this study, with 4,018 primiparas ultimately included. comprising 914 cases in the massage group and 3,104 in the control group before matching. Significant differences were observed between the two groups in maternal age, educational level, and analgesic method (*P* < 0.05). After PSM, 1,828 participants were retained, with 914 women in each group. PSM showed that baseline characteristics were well balanced, with no significant differences in maternal height, pre-pregnancy weight, maternal age, gestational weight gain, weeks of gestation, neonatal weight, educational level, analgesic method (epidural or non-epidural), mode of delivery, or neonatal weight (*P* > 0.05). These results indicate good comparability between the two groups. Detailed data are presented in Table 1.

**Table 1:** Basic data before and after matching

		Before matching				After matching			
Variables	Grouping	Control group (*n* = 3104)	Massage group (*n* = 914)	t/Z/cardinal value	*P*	Control group (*n* = 914)	Massage group (*n* = 914)	t/Z/cartesian value	*P*
Maternal height		163.28 ± 11.11	162.83 ± 5.00	1.176	0.240	162.82 ± 10.85	162.82 ± 5.00	0.003	0.998
Pre-pregnancy weight		55.27 ± 10.91	55.57 ± 7.52	−0.769	0.442	55.43 ± 11.35	55.58 ± 7.53	−0.320	0.749
Maternal age		29.14 ± 3.15	30.19 ± 2.79	−9.093	< 0.001	30.07 ± 3.06	30.18 ± 2.77	−1.020	0.308
Week of gestation		39.00 ± 1.04	38.95 ± 0.89	1.389	0.165	38.95 ± 1.08	38.95 ± 0.89	0.118	0.906
Gestational weight gain		12.63 ± 3.12	12.61 ± 4.27	0.132	0.895	12.54 ± 3.18	12.60 ± 4.27	−0.380	0.704
Educational level	College and below	1071(34.5%)	110 (12.0%)	5.921	< 0.001	117(12.8%)	110(12.0%)	0.833	0.405
	Undergraduate	1047(33.7%)	559(61.2%)			558(61.2%)	559(61.1%)		
	Postgraduate	986(31.8%)	245(26.8%)			239(26.0%)	245(26.9%)		
Analgesic method	non-epidural	891(28.7%)	182(19.9%)	27.888	< 0.001	188(20.5%)	182(20.0%)	0.059	0.808
	epidural	2213(71.3%)	732 (80.1%)			726(79.5%)	732(80.0%)		
Mode of delivery	spontaneous delivery	2870(92.5%)	848(92.8%)	0.005	0.943	829(90.7%)	848(92.8%)	0.072	0.788
	Assisted vaginal delivery	234(7.5%)	66 (7.2%)			85 (9.3%)	66 (7.2%)		
Neonatal weight		3210.87 ± 343.52	3234.89 ± 321.48	−1.885	0.059	3220.97 ± 350.64	3234.89 ± 321.48	−0.884	0.377

### Comparison of labor outcomes between the two groups after matching

#### Comparison of labor outcomes

The difference in the distribution of perineal injuries between the two groups was statistically significant (*χ²*=15.950, *P* = 0.002). The rate of perineal integrity was higher in the massage group than in the control group (2.2% vs. 0.5%), while the episiotomy rate was lower (16.3% vs. 21.2%). The control group showed a slightly lower rate of first-degree lacerations (53.5% vs. 58.6%), whereas the massage group had a lower rate of second-degree lacerations (22.9% vs. 24.7%). Severe perineal lacerations (Grade III/IV) were rare in both groups, with none observed in the massage group and one case in the control group. The second stage of labor was significantly shorter in the massage group compared with the control group (1.27 h vs. 1.42 h, *P* = 0.001). No significant differences were found between groups in the duration of the first stage (9.39 h vs. 9.50 h), third stage (0.12 h vs. 0.10 h), or total labor duration (11.08 h vs. 11.17 h) (*P* > 0.05). Detailed results are presented in Table 2.

**Table 2:** Comparative results of labor outcome indicators

Indicator	Group	Control group (*n* = 914)	Massage group (*n* = 914)	t/Z/cardinal value	*P*
Mode of delivery	Spontaneous delivery	829 (90.7%)	848 (92.8%)	0.072	0.788
	Assisted vaginal delivery	85 (9.3%)	66 (7.2%)		
Perineal condition	Intact	5(0.5%)	20(2.2%)	15.950	0.002
	Degree I	488(53.5%)	536(58.6%)		
	Degree II	226(24.7%)	209(22.9%)		
	III/IV	1(0.1%)	0(0.0%)		
	Lateral cut	194(21.2%)	149(16.3%)		
Duration of Labor and Delivery	First stage of labor	9.50 (6.68,12.56)	9.39(6.50,12.52)	−0.358	0.720
	Second stage of labor	1.42(0.85,2.14)	1.27 (0.72,1.93)	−3.472	0.001
	Third stage of labor	0.10(0.07, 0.13)	0.12(0.08, 0.15)	0.102	0.089
	Total length of labor	11.17(8.33,14.42)	11.08(7.92,14.35)	−0.845	0.398
Neonatal weight		3220.97 ± 350.64	3234.89 ± 321.48	−0.884	0.377

### Analysis of influencing factors of variables with differences

#### Analysis of factors influencing perineal injury

Univariate analysis showed no significant differences in educational level, BMI, maternal age, weeks of gestation, or gestational weight gain among groups with varying degrees of perineal injury (intact, first-degree, and second-degree or higher) (*P* > 0.05). A significant difference was observed in neonatal weight (*P* = 0.032), with the highest mean weight in the group experiencing second-degree or higher lacerations (3264.54 ± 309.13 g). Mode of delivery had a significant effect on perineal integrity (*P* < 0.001), with the intact perineum rate being markedly higher in spontaneous deliveries compared to assisted vaginal deliveries, where 93.93% of cases involved second-degree or higher lacerations. The number of massage sessions also showed a significant gradient across injury groups (*P* < 0.001). The median number of sessions was highest in the intact perineum group (7, IQR: 3–8), followed by the first-degree laceration group (2, IQR: 1–4) and the second-degree or higher group (1, IQR: 1–2). Detailed results are presented in Table 3.

**Table 3:** Results of single factor analysis

Indicator	Subgroup	Intact (*n* = 20)	first-degree (*n* = 535)	second-degree or higher (*n* = 359)	Calculation/F	*P*
BMI		20.88 ± 2.41	20.99 ± 2.62	20.88 ± 2.46	0.218	0.804
Maternal age		30.05 ± 3.34	30.11 ± 2.75	30.31 ± 2.82	0.581	0.559
Weeks of gestation		39.26 ± 0.73	38.93 ± 0.92	38.96 ± 0.86	1.347	0.261
Gestational weight gain		11.52 ± 4.48	12.78 ± 4.37	12.42 ± 4.10	1.407	0.245
Mode of delivery (914)	Spontaneous delivery	20 (2.35%)	531 (62.63%)	297 (35.02%)	89.133	<0.001
	Assisted vaginal delivery	0 (0.0%)	4(6.07%)	62(93.93%)		
Neonatal weight		3117.89 ± 329.52	3219.18 ± 327.79	3264.54 ± 309.13	3.443	0.032
Number of Massages		7.00(3.00,8.00)	2.00(1.00,4.00)	1.00(1.00,2.00)	61.013	<0.001

Multivariable ordered logistic regression analysis showed that the number of massages was significantly and negatively associated with the severity of perineal injury (*OR* = 0.723, 95%*CI*: 0.670–0.780, *P* < 0.001), indicating that each additional massage reduced the severity of perineal injury by 27.7%. The risk of perineal injury increased slightly with each 1 g increase in neonatal weight (*OR* = 1.001,*P* = 0.006). The risk of severe perineal injury was 34.8 times higher in assisted vaginal deliveries than in spontaneous deliveries (*OR* = 34.815, 95%*CI*: 11.960–101.343, *P* < 0.001), as presented in Table 4.

**Table 4:** Results of multifactorial ordered logistic regression analysis

Indicator	B	Standard error	Wald	*P*	OR	95% CI	
						Lower limit	Upper limit
Number of massages	−0.324	0.039	70.215	<0.001	0.723	0.670	0.780
Neonatal weight	0.001	0.000	7.432	0.006	1.001	1.000	1.001
Mode of delivery	3.550	0.545	42.408	<0.001	34.815	11.960	101.343

Parallelism test, chi-square value = 3.369, *P* = 0.338

#### Analysis of factors influencing the second stage of labor

Spearman correlation analysis indicated no significant correlation between BMI, maternal age, weeks of gestation, or gestational weight gain and the duration of the second stage of labor (*P* > 0.05). Only the number of massages showed a significant negative correlation with the length of the second stage of labor (*rs* = − 0.303, *P* < 0.001), as shown in Table 5.

**Table 5:** Results of single factor analysis

Indicator	Second stage of labor	
	rs	*P*
BMI	0.009	0.781
Maternal age	−0.010	0.755
Weeks of gestation	0.006	0.847
Gestational weight gain	0.007	0.829
Neonatal weight	0.058	0.081
Number of massages	−0.303	<0.001

Multiple linear regression analysis demonstrated that the number of massages had an independent effect on the duration of the second stage of labor (*B* = − 0.123, *P* < 0.001), indicating that each additional massage shortened this stage by approximately 0.123 h (about 7.4 min). The mode of delivery also had a significant impact, with assisted vaginal deliveries lasting 0.522 h longer than spontaneous deliveries (*P* < 0.001), as shown in Table 6.

**Table 6:** Results of multiple linear regression analysis

Indicator	Unstandardized coefficient		Standardized coefficient	t	*P*	B 95.0% CI		Covariance statistics	
	B	Standard error	Beta			Lower	Upper limit	Tolerance	VIF
(Constant)	1.439	0.082		17.453	<0.001	1.277	1.601		
Number of massages	−0.123	0.014	−0.275	−8.832	<0.001	−0.151	−0.096	0.991	1.009
Mode of delivery	0.522	0.122	0.133	4.290	<0.001	0.283	0.760	0.996	1.004

*F* = 44.660,*P* < 0.001,*R2* = 0.128

### Comparison of safety indicators

Postpartum hemorrhage was lower in the massage group compared with the control group (293 ± 130 ml vs. 306 ± 138 ml, *P* = 0.038), and the incidence of premature rupture of membranes was also reduced (22.4% vs. 27.2%, *χ²* = 7.567, *P* = 0.006). No significant differences were observed between the groups in the incidence of preterm labor or in the 1-minute and 5-minute neonatal Apgar scores (*P* > 0.05), as shown in Table 7.

**Table 7:** Comparative results of safety indicators

Indicator	Group	Control group (*n* = 914)	Massage group (*n* = 914)	t/cardinal value	*P*
Postpartum hemorrhage		306.39 ± 138.36	293.30 ± 130.87	2.078	0.038
Premature rupture of membranes	No	665 (72.8%)	709 (77.6%)	7.567	0.006
	Yes	249(27.2%)	205(22.4%)		
Preterm labor	No	892(97.6%)	902(98.7%)	1.471	0.225
	Yes	22(2.4%)	12(1.3%)		
1-minute assessment		9.93 ± 0.46	9.92 ± 0.50	0.439	0.661
5-minute assessment		9.99 ± 0.19	9.98 ± 0.26	0.311	0.755

## Discussion

### Effect of perineal massage on labor outcomes

This study found that the rate of perineal integrity was significantly higher in the massage group than in the control group, while the rates of episiotomy and second-degree laceration were lower. The second stage of labor was shortened by an average of nine minutes, consistent with the findings of Lin Mei-Zhi et al. [10], who reported an average reduction of 6.3 min. Similarly, Wang Wen-Juan et al. [11] indirectly confirmed the effectiveness of perineal massage in late pregnancy by combining it with pelvic training, demonstrating reductions in both the severity of perineal injury and the duration of the second stage of labor. Yin J et al. [6] compared perineal massage during the second stage of labor with that performed in late pregnancy and found that late -pregnancy massage significantly reduced the risk of second-degree or higher lacerations (*RR* = 0.69, 95%*CI* [0.53–0.90]) and decreased the episiotomy rate by approximately 15%. A foreign [5] study involving 3,374 women reported similar results, suggesting that regular perineal massage enhances tissue elasticity, promotes local relaxation, and improves blood circulation. Moreover, the interaction between midwives and parturients during massage may alleviate tension and facilitate better labor coordination, thereby shortening labor duration and reducing perineal trauma [8]. However, delivery outcomes are influenced by multiple complex factors, including social conditions [12], maternal physical and psychological states [13], and fetal health [14]. Therefore, clinical practice should consider the individual preferences and circumstances of pregnant women, implementing personalized perineal massage protocols to optimize delivery outcomes.

### The number of perineal massage is a protective factor associated with reduced perineal injury and a shorter second stage of labor

Further analysis within the massage group revealed a highly significant difference in the number of massages among participants with varying degrees of perineal injury (*P* < 0.001). The median number of massages in the intact perineum group was 7 (IQR: 3–8), significantly higher than in the first-degree laceration group (2, IQR: 1–4) and the second-degree or higher laceration group (1, IQR: 1–2), suggesting that increasing the number of massages may be a key factor in reducing the risk of perineal injury. Multivariable ordered logistic regression analysis further confirmed a significant negative association between the number of massages and the severity of perineal injury (*OR* = 0.723, 95% *CI*: 0.670–0.780, *P* < 0.001), indicating that each additional massage reduced the severity of perineal injury by approximately 27.7%. This relationship appeared nonlinear, with optimal perineal protection achieved at around seven massage sessions. In the analysis of factors influencing the duration of the second stage of labor, univariate results showed a significant negative correlation between the number of massages and labor duration (Spearman’s *ρ* = −0.303, *P* < 0.001), suggesting that a higher frequency of perineal massage was associated with a shorter second stage of labor. Multiple linear regression confirmed this relationship, showing that the number of massages remained an independent factor influencing labor duration (*β*=−0.123 h/session, 95% *CI*: −0.182 to − 0.064, *P* < 0.001), meaning that each additional massage shortened the second stage of labor by approximately 7.4 min. This effect may result from regular massage promoting the orderly alignment of collagen fibers in the perineal tissues, enhancing their elasticity and reducing resistance during delivery [5]. Additionally, stimulation of pelvic floor proprioceptors through massage may improve the efficiency of voluntary exertion, alleviate fear of labor, and enhance maternal cooperation [15, 16]. Although this study demonstrated a significant association between the number of massages, the severity of perineal injury, and the duration of the second stage of labor, the specific dose-response relationship requires further investigation.

### Safety evaluation of perineal massage

The results of this study showed that there was no severe (Grade III/IV) perineal laceration occurred in the massage group, while one case was observed in the control group. Although the difference was not statistically significant, the finding suggests that perineal massage does not increase the risk of severe perineal trauma. Notably, certain maternal and obstetric characteristics may predispose women to high-grade perineal injuries, highlighting the clinical importance of targeted preventive strategies. A recent case–control study by Aquino et al. [17], which included 116 cases of severe perineal laceration and 348 controls, identified fetal macrosomia, operative vaginal delivery, and prolonged second stage of labor as significant risk factors. These findings emphasize the potential value of individualized preventive measures, such as perineal massage, particularly for women at higher risk. In this context, the absence of severe lacerations in the massage group may reflect not only the safety of the intervention but also its potential protective effect in populations with elevated baseline risk.

Postpartum hemorrhage in the massage group was 13 ml lower than in the control group, which may be attributed to the ability of perineal massage to promote local microcirculation, enhance tissue elasticity, and thereby reduce the risk of birth canal injury. In a qualitative study exploring barriers to the implementation of perineal massage among pregnant women in late pregnancy, Haley Tang [18] et al. reported that some women expressed concerns that mechanical stimulation during massage might increase the risk of premature rupture of membranes, infection, or preterm labor. Similar perceptions were also observed among Thai pregnant women [19]. However, the present study found that the incidence of premature rupture of membranes was lower in the massage group than in the control group, suggesting that perineal massage does not increase this risk. Further analysis indicated that strict adherence to hand hygiene and the use of medical lubricants can effectively prevent pathogen invasion [16, 19]. Moreover, perineal massage may enhance maternal self-care awareness, indirectly reducing infection risk. Given the well-established association between premature rupture of membranes and infectious episodes [20], these findings suggest that perineal massage does not increase the risk of fetal infection.

There were no significant differences between the two groups in the incidence of preterm labor or neonatal Apgar scores. In addition, review of the perineal massage records by the midwives’ clinic revealed no serious adverse events such as increased pain, vaginal discharge, or bleeding during or after massage. These findings collectively indicate that, when performed under standardized procedures, perineal massage does not adversely affect intrauterine development or neonatal outcomes and can be considered a safe intervention during late pregnancy.

### Study significance

The Clinical Practice Guidelines for the Prevention and Management of Perineal Laceration in Vaginal Delivery recommend initiating perineal massage at 34 weeks of gestation for women planning vaginal delivery and without contraindications to it [2]. In this study, considering maternal acceptance and the relative fetal maturity at 36 weeks, perineal massage was initiated at 36 weeks of gestation and performed two to three times per week until delivery. However, statistical analysis revealed that the average number of massage sessions was only 2.75 per participant (range 0–12), indicating insufficient frequency and poor compliance. This may be attributed to limited time availability, the short intervention window, safety and efficacy concerns, and the overall low adoption rate. Using a large cohort and applying PSM to control for confounding variables, this study confirmed the effectiveness and safety of perineal massage in late pregnancy for improving delivery outcomes. Increasing the frequency of perineal massage was identified as a protective factor associated with reduced perineal trauma and a shorter second stage of labor. Therefore, midwife clinics should play an active role in promoting and supervising perineal massage, tailoring interventions to individual circumstances and preferences. Encouraging women to achieve the median of seven sessions observed in the intact perineum group may ensure sufficient cumulative intervention to optimize perineal elasticity, enhance birth canal compliance, reduce perineal injury, and shorten labor duration. In addition, incorporating perineal massage education into prenatal care can help disseminate knowledge about childbirth, standardize weight management during pregnancy, teach midwifery techniques, familiarize women with labor processes, alleviate fear of childbirth, and ultimately support safe and successful vaginal delivery.

## Strengths and limitations of the study

This study employed the PSM method to effectively control confounding bias in examining the effects of perineal massage during late pregnancy by balancing baseline characteristics such as age, height, weight, weeks of gestation, and gestational weight gain through 1:1 matching. With an adequate sample size, the study was able to evaluate both the clinical efficacy and safety of the intervention and to recommend a specific frequency of perineal massage (2–3 sessions per week, ≥ 7 times cumulatively) for clinical practice. However, the number of massages remains influenced by the timing of labor and delivery, and the specific dose–response relationship requires further validation through prospective research. As a retrospective, single-center observational study, the findings may have limited generalizability. Additionally, this study did not assess psychological factors such as anxiety and depression, pelvic floor muscle function, or inter-observer variability, which may influence delivery outcomes. Future multicenter randomized controlled trials incorporating standardized psychological and pelvic floor function assessments are needed to validate the optimal frequency of perineal massage and further clarify its clinical value.

## Data Availability

The datasets generated and/or analyzed during the present study are available from the corresponding author upon reasonable request.

## Abbreviations

PSM: Propensity Score Matching
EMRS: Electronic Medical Record System
RCOG: Royal College of Obstetricians and Gynaecologists
OR: Odds Ratio
CI: Confidence Interval
